# Functional Interplay Between Upper Loop Reentry and Clockwise Cavotricuspid Isthmus-Dependent Flutter in a Non-surgical Right Atrium: A Case of Dual-Loop Macroreentry

**DOI:** 10.7759/cureus.105469

**Published:** 2026-03-18

**Authors:** Cristian J Sosa-Alvarez, Daniela Cordoba-Alvarado, Oriana Y Mariscal-Diaz, Jorge A Loza-Nuño

**Affiliations:** 1 Cardiology, Instituto de Seguridad y Servicios Sociales de los Trabajadores del Estado, Zapopan, MEX; 2 Internal Medicine, Secretaria de Salud, Zapopan, MEX; 3 Electrophysiology, Instituto de Seguridad y Servicios Sociales de los Trabajadores del Estado, Zapopan, MEX

**Keywords:** atrial flutter, cavotricuspid isthmus, dual-loop macroreentry, electroanatomical mapping, upper loop reentry

## Abstract

Typical atrial flutter is a common macroreentrant arrhythmia dependent on the cavotricuspid isthmus (CTI). However, complex right atrial substrates can support multiple concurrent reentrant circuits even in the absence of prior cardiac surgery. We present the case of a 68-year-old man with persistent clockwise atrial flutter and no history of surgical intervention or prior ablation. High-density electroanatomical mapping and entrainment maneuvers identified a dual-loop macroreentrant mechanism involving both a clockwise CTI-dependent circuit and an upper loop reentry circuit. Initial ablation of the superior loop resulted in a significant prolongation of the tachycardia cycle length from 264 ms to 308 ms without termination, unmasking the slower CTI-dependent component. Restoration of sinus rhythm was achieved only after targeted ablation of fragmented potentials at the CTI-inferior vena cava junction and completion of a bidirectional CTI block. This case highlights the functional interplay between different right atrial loops facilitated by functional barriers like the crista terminalis in a non-surgical atrium. It underscores the importance of systematic mapping and the recognition that CTI ablation alone may be insufficient when multiple loops coexist.

## Introduction

Typical atrial flutter is defined as a macroreentrant atrial tachycardia critically dependent on conduction through the cavotricuspid isthmus (CTI). The most common activation pattern is a counterclockwise rotation around the tricuspid annulus; however, the same reentrant circuit may occur in a clockwise direction, sharing identical anatomic boundaries but with an opposite activation sequence [1].

In contrast, atypical atrial flutter encompasses a heterogeneous group of macroreentrant atrial tachycardias that are not dependent on the CTI. The arrhythmogenic substrate usually results from a combination of fixed anatomical structures, such as valves or venous orifices, and areas of atrial scar related to prior catheter ablation or cardiac surgery. Nevertheless, atypical flutter may also occur in patients without previous interventions or evident structural atrial remodeling [2].

Upper loop reentry represents an uncommon form of right atrial macroreentrant tachycardia in which the reentrant circuit is located in the superior portion of the right atrium, typically involving the region around the superior vena cava and the crista terminalis, and does not critically depend on the CTI. In this mechanism, activation of the remaining atrial myocardium occurs passively. Identification of upper loop reentry requires detailed electrophysiological assessment, often relying on conventional pacing maneuvers and three-dimensional electroanatomic mapping to delineate atrial activation patterns, areas of conduction block, and zones of slow conduction [3].

Recognition of this mechanism can be particularly challenging when it coexists with typical atrial flutter, as surface electrocardiographic features may be misleading and CTI ablation alone may be insufficient to terminate the arrhythmia [4].

## Case presentation

**Figure 1:**
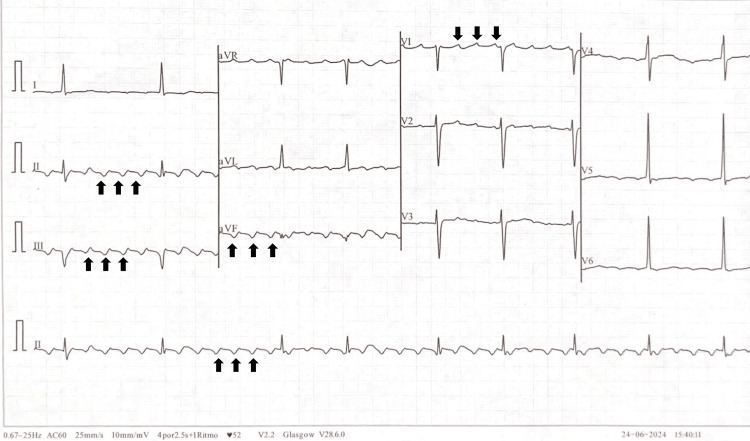
Surface electrocardiogram showing atrial flutter. Black arrows indicate the characteristic predominantly negative f waves in the inferior leads (II, III, and aVF, where arrows point up to negative deflections) and predominantly positive f waves in lead V1 (arrows point down to positive peaks).

A 68-year-old man with a history of hypertension, dyslipidemia, benign prostatic hyperplasia, and hip osteoarthritis was referred to our center for the management of persistent atrial flutter. The patient reported intermittent palpitations. Six years prior, during a preoperative assessment for cholecystitis, he had been diagnosed with clockwise typical atrial flutter (Figure 1).

A previous attempt at electrical cardioversion had been unsuccessful. Notably, the patient had no history of prior cardiac surgery or catheter ablation. Upon admission, the patient’s vital signs were as follows: blood pressure 130/78 mmHg, heart rate 72 bpm, respiratory rate 17 breaths per minute, body temperature 36.7°C, and peripheral oxygen saturation (SpO_2_) 94% on room air.

**Figure 2:**
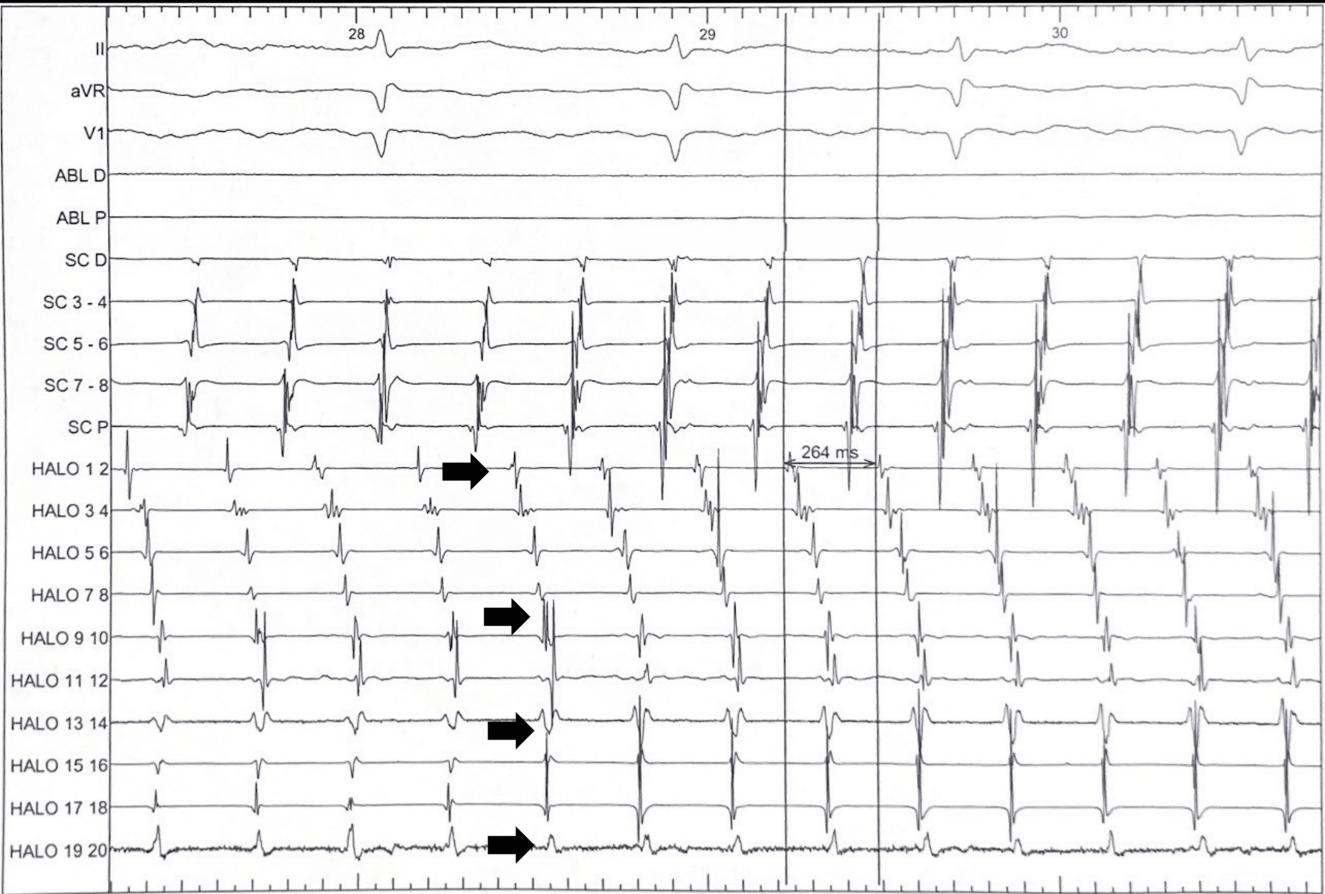
Intracardiac electrograms demonstrating activation sequence. The tracing displays surface ECG leads (II, aVR, V1) and intracardiac recordings from the ablation (ABL), CS, and duodecapolar lateral right atrial (HALO) catheters. Black arrows highlight the sequential atrial activation within a single tachycardia cycle, illustrating a craniocaudal (proximal-to-distal) sequence along the HALO catheter (from HALO 1-2 to HALO 19-20). This activation pattern is consistent with a clockwise cavotricuspid isthmus-dependent atrial flutter. The tachycardia cycle length is stable at 264 ms (indicated by vertical lines). CS: coronary sinus; ECG: electrocardiogram.

The procedure was performed under conscious sedation. The baseline rhythm was sustained atrial flutter (AFL) with a clockwise activation pattern and a tachycardia cycle length (TCL) of 264 ms. Multipolar catheters were positioned in the coronary sinus (10-pole) and along the lateral wall of the right atrium (20-pole duodecapolar) (Figure 2).

**Figure 3:**
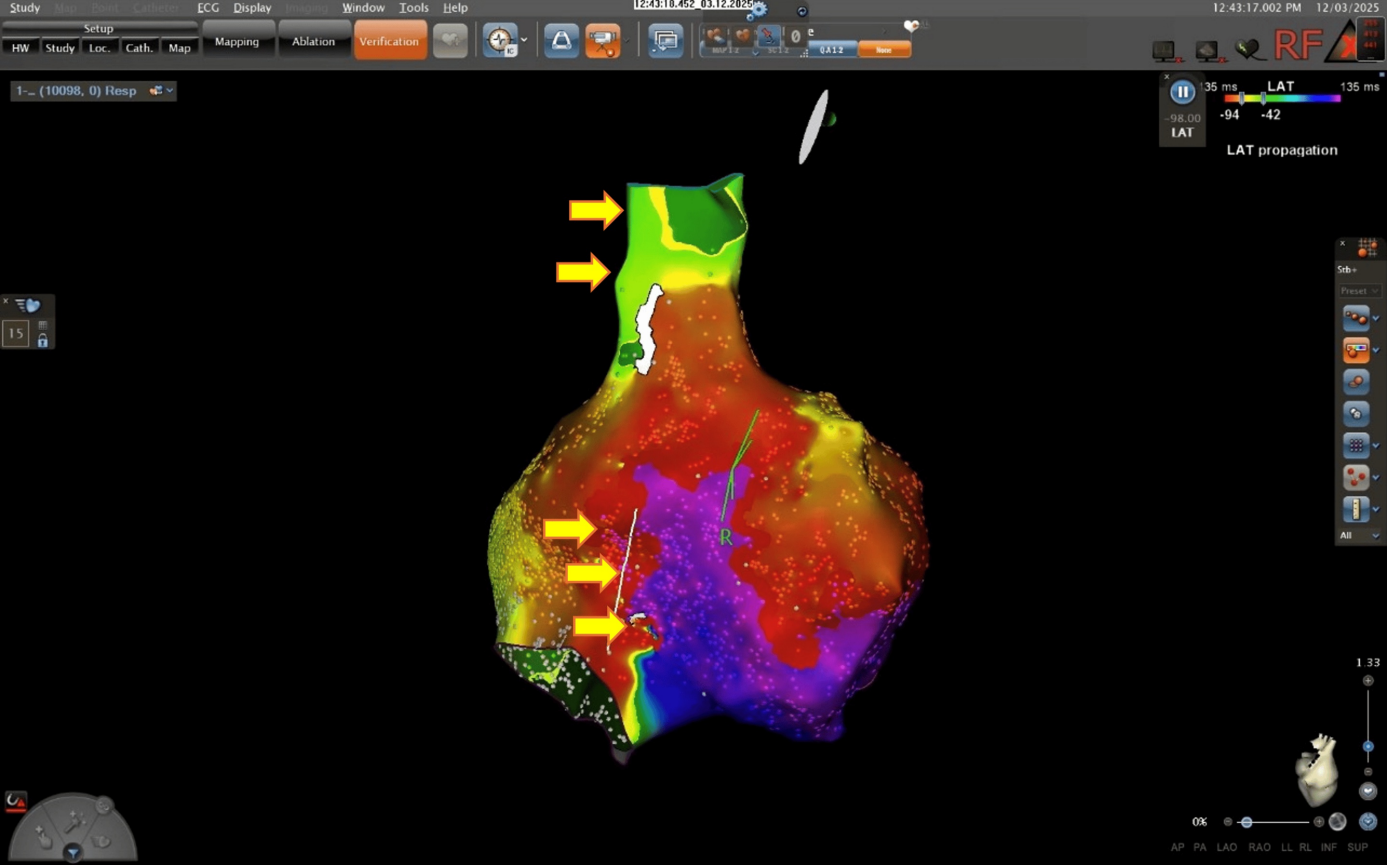
Electroanatomical local activation time map. Electroanatomical local activation time map in the left anterior oblique projection using the CARTO® 3 electroanatomical mapping system (Biosense Webster, Irvine, CA, USA), demonstrating a dual-loop macroreentrant pattern. Yellow arrows indicate the two concurrent wavefronts: a typical CTI-dependent circuit and a superior right atrial loop encircling the superior vena cava. The "early-meets-late" zone is clearly visualized, confirming the macroreentrant nature of the arrhythmia.

**Figure 4:**
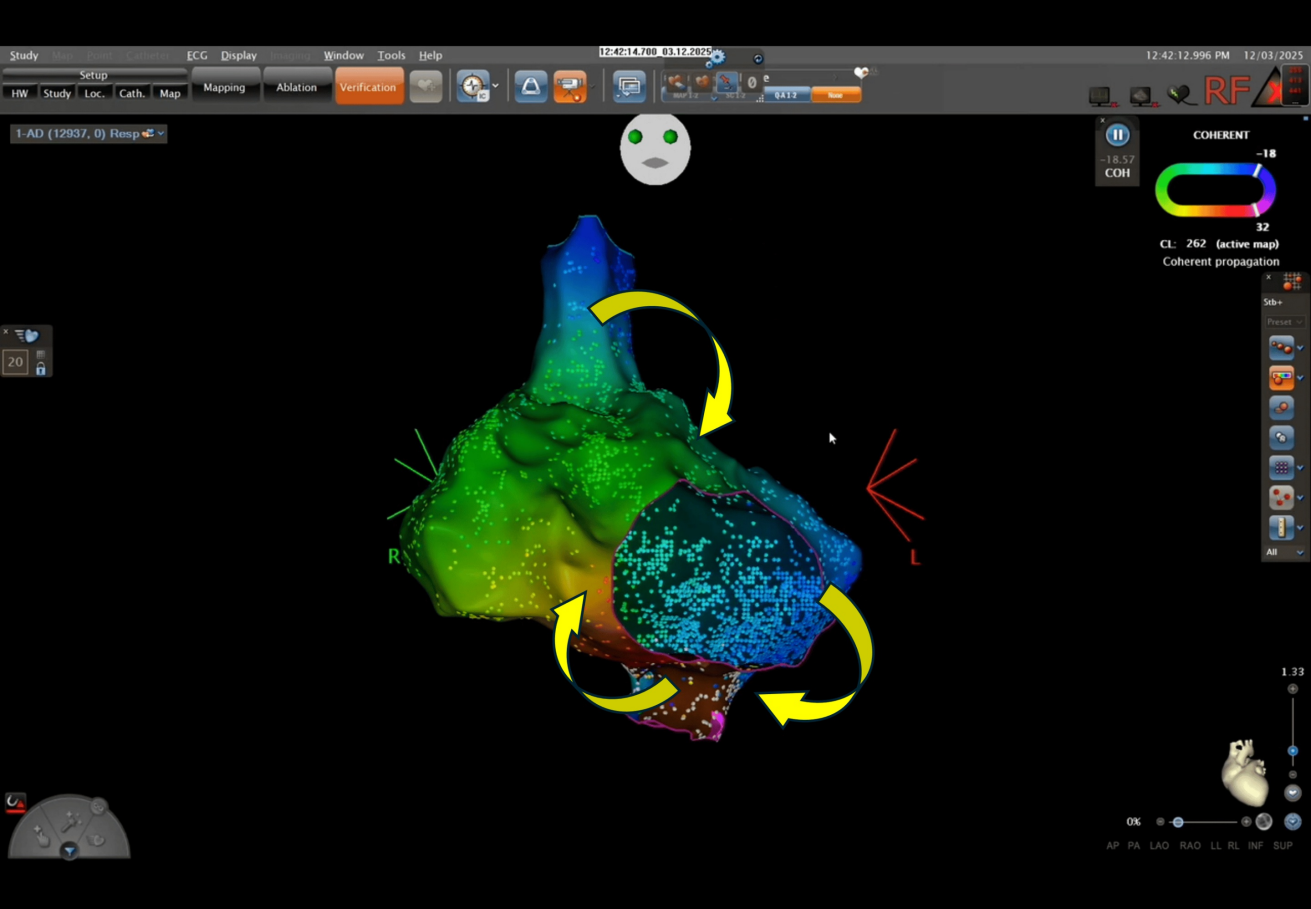
Three-dimensional electroanatomical clockwise activation map. Activation map generated using the CARTO® 3 system (Biosense Webster). Yellow arrows indicate the clockwise activation front around the tricuspid annulus. A clear conduction bottleneck is visualized at the cavotricuspid isthmus (CTI). The transition from the late activation zone (purple) back to the early activation zone (red/orange) confirms the reentrant mechanism.

A high-density three-dimensional electroanatomical map (CARTO3) was created using a multielectrode mapping catheter (Pentaray; Biosense Webster, Irvine, CA, USA). The activation mapping suggested participation of both the CTI (Figure 3) and a superior right atrial loop within a complex macroreentrant pattern (Figure 4).

High-density voltage mapping revealed areas of low voltage (<0.5 mV) and fragmented electrograms in the posterior right atrial wall, suggesting an idiopathic fibrotic substrate. Entrainment pacing was performed from both the CTI and the posterior wall to delineate the circuits. The CTI region yielded a post-pacing interval (PPI) minus TCL of 30 ms, confirming its critical involvement.

**Figure 5:**
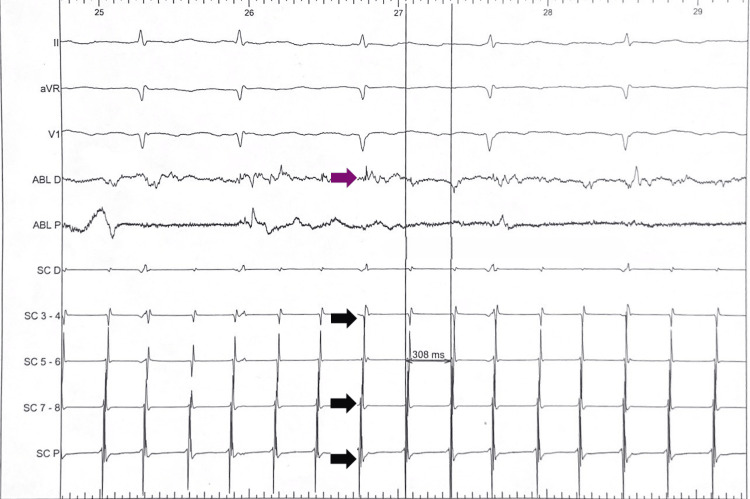
Intraprocedural prolongation of the tachycardia cycle length from 264 ms to 308 ms during radiofrequency ablation, as recorded by the LabSystem™ Pro (Boston Scientific), indicating modification of the superior loop component without arrhythmia termination. The purple arrow indicates the site of radiofrequency application at the ablation catheter (ABL D), leading to circuit modification. Black arrows illustrate the maintained atrial activation sequence along the coronary sinus catheter despite the cycle length increase.

Ablation was performed using a contact-force sensing catheter (SmartTouch). Radiofrequency (RF) delivery was initially targeted at the posterior right atrial circuit, extending a lesion toward the superior vena cava. This intervention resulted in a sudden prolongation of the TCL from 264 ms to 308 ms (Figure 5).

**Figure 6:**
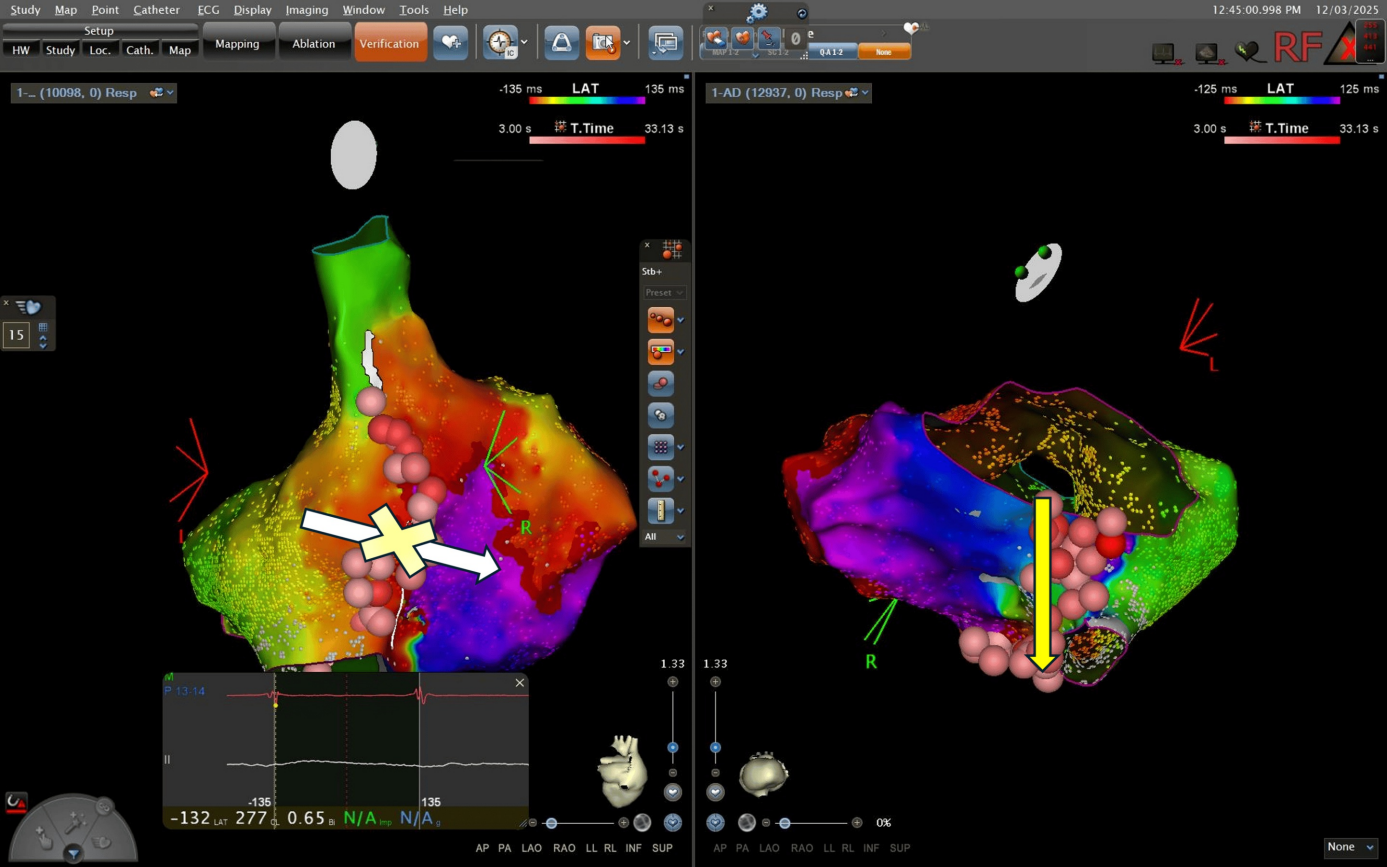
Post-ablation cavotricuspid isthmus block verification. Electroanatomical activation map (RAO projection) generated using the CARTO® 3 system. The yellow "X" over the white arrow visually indicates complete, bidirectional block at the cavotricuspid isthmus, where the activation wavefront is stopped. The vertical yellow arrow highlights the continuous radiofrequency ablation line (red points) responsible for terminating the reentrant circuit. RAO: right anterior oblique.

This 44 ms shift without arrhythmia termination indicated the elimination of the faster superior loop circuit, unmasking the slower clockwise CTI-dependent flutter. Subsequently, a linear lesion was completed at the CTI from the tricuspid annulus to the inferior vena cava (IVC) at 35 W (Figure 6).

**Figure 7:**
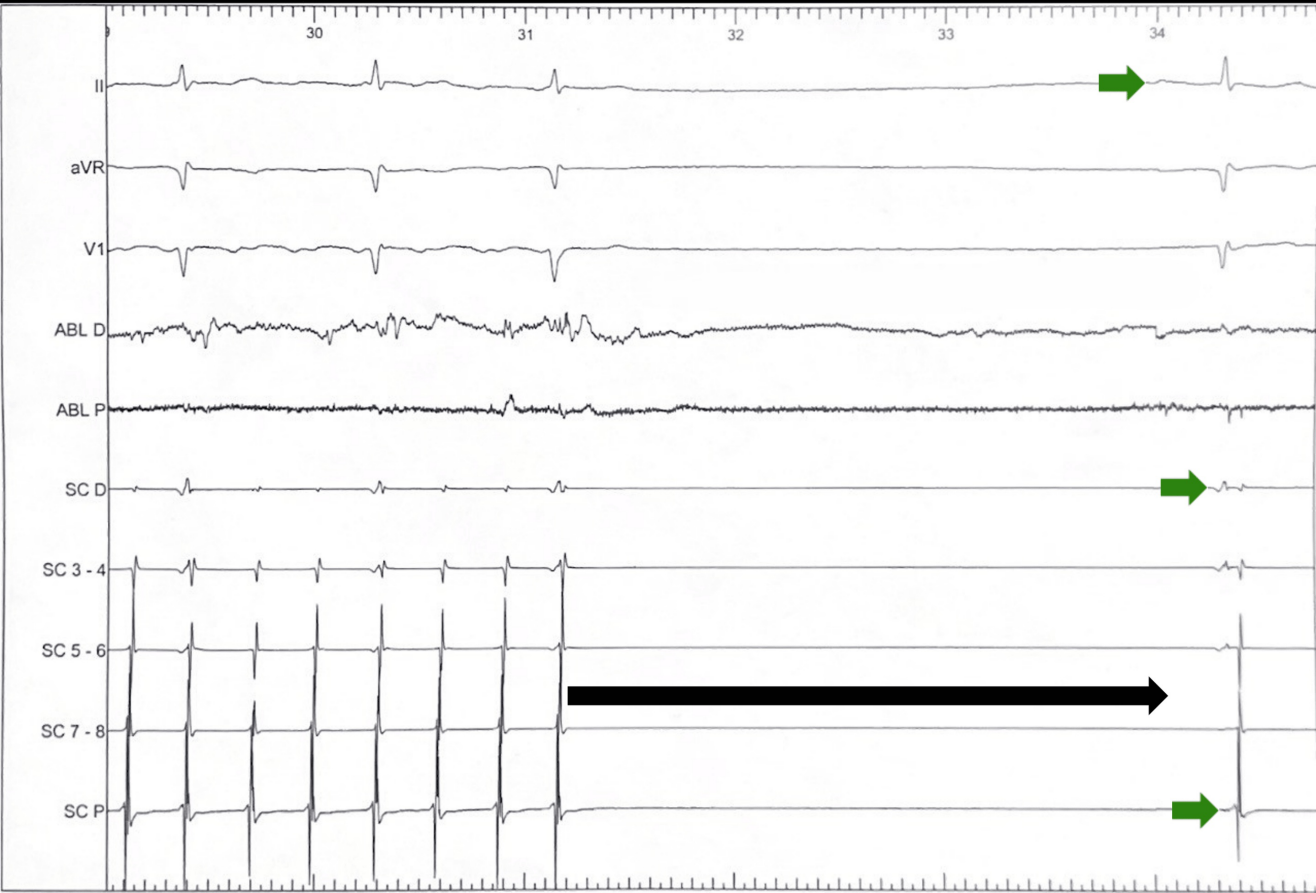
Acute termination of atrial flutter to sinus rhythm during CTI ablation, confirmed by intracardiac recordings and surface ECG obtained with the LabSystem™ Pro (Boston Scientific). The long black arrow clearly indicates the precise moment of tachycardia cessation and the subsequent period of silence. The green arrows mark the restoration of organized sinus rhythm, confirmed by surface ECG and organized intracardiac signals in the CS and ablation (ABL) catheters. CS: coronary sinus; CTI: cavotricuspid isthmus; ECG: electrocardiogram.

Despite the line completion, the AFL persisted at 308 ms. A targeted remap identified residual fragmented potentials near the IVC-isthmus junction. Brief RF delivery at this specific site resulted in the abrupt termination of the arrhythmia and restoration of sinus rhythm (Figure 7).

**Figure 8:**
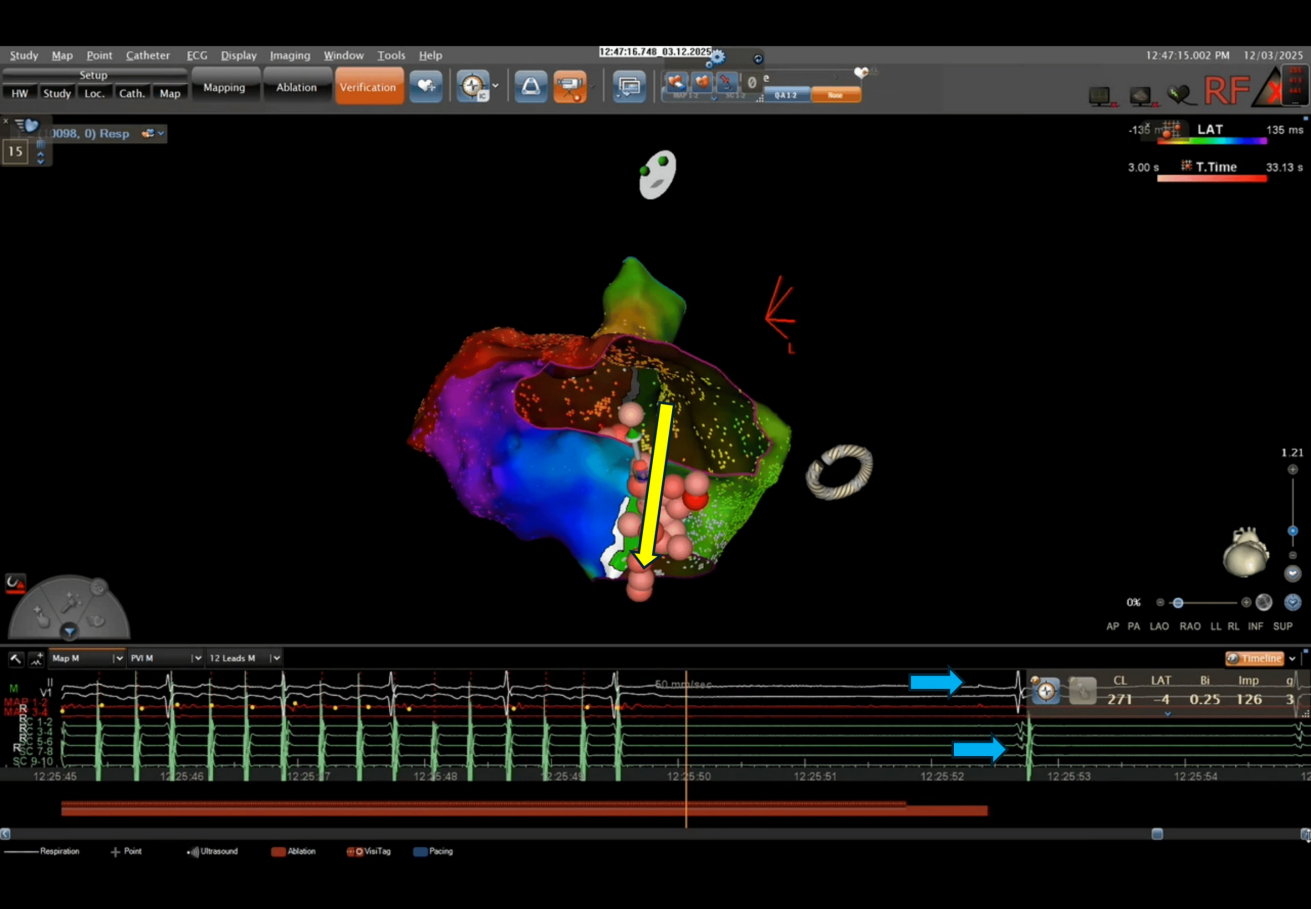
Final ablation site at the CTI–IVC junction demonstrating abrupt termination of atrial flutter and confirmation of bidirectional CTI block by differential pacing maneuvers using the CARTO® 3 system. Diagonal yellow arrow pointing towards the precise CTI location where successful arrhythmia termination was achieved. Lower panels display real-time electrogram recordings; vertical blue arrows indicate the organized return of stable sinus rhythm, confirmed by simultaneous surface ECG and organized intracardiac signals. CTI: cavotricuspid isthmus; IVC: inferior vena cava; ECG: electrocardiogram.

Bidirectional CTI block was confirmed via differential pacing, with clockwise and counterclockwise conduction times of 168 ms and 145 ms, respectively (Figure 8).

Final intracardiac intervals were within normal limits (PR 210 ms, AH 68 ms, HV 48 ms), and the patient remained in sinus rhythm at the end of the procedure. 

## Discussion

Typical atrial flutter is generally considered a straightforward arrhythmia, with high success rates following cavotricuspid isthmus ablation. However, this case illustrates that additional macroreentrant circuits may coexist within the right atrium, leading to persistence of the arrhythmia despite the creation of a complete CTI line. Previous studies have demonstrated that more than one reentrant mechanism can be present simultaneously, and that entrainment mapping is essential to identify all components of the tachycardia circuit [5].

In typical atrial flutter, the reentrant wavefront circulates around the tricuspid annulus, with the cavotricuspid isthmus acting as the critical slow-conduction zone. Anatomical structures such as the tricuspid annulus and the crista terminalis contribute to the stabilization of this circuit by forming lines of conduction block. Even in structurally normal atria, transverse conduction across the crista terminalis is limited due to marked anisotropy, favoring longitudinal conduction and functional block. Importantly, these electrophysiological properties may permit the development of additional reentrant circuits in the superior right atrium [6].

Although the coexistence of typical flutter and superior atrial reentry circuits has been classically described in patients with prior atrial surgery, functional barriers in the superior right atrium may also allow the presence of upper loop reentry in non-surgical atria [7], as illustrated by the present case. This case highlights that in nonsurgical atria, functional barriers, primarily the crista terminalis, can facilitate the coexistence of typical CTI-dependent flutter and upper loop reentry. The observation that CTI ablation alone did not terminate the arrhythmia, whereas upper loop modification significantly prolonged the cycle length (from 264 to 308 ms), underscores the functional interplay between these two circuits. The final termination at the CTI-IVC junction, following a high-density remap, suggests that even in the presence of superior RA reentry, the isthmus often remains a critical bottleneck or part of a common pathway for both wavefronts. The abrupt 44 ms prolongation in tachycardia cycle length without termination indicates modification of the overall circuit path length rather than elimination of an independent tachycardia, supporting the presence of a functionally coupled macroreentrant mechanism.

Furthermore, it is crucial to consider the broader context of cardiovascular care delivery. Significant disparities persist in the prevalence of cardiovascular risk factors and access to specialized interventions like catheter ablation. Data indicate that non-Hispanic Black and Hispanic populations face a disproportionate burden of hypertension and diabetes. These disparities are often compounded by lower socioeconomic status, including limited educational attainment, higher unemployment rates, and a lack of adequate insurance coverage. Such systemic barriers not only worsen cardiovascular outcomes but also limit access to advanced electrophysiological procedures in vulnerable communities [8].

## Conclusions

The occurrence of dual-loop macroreentry in patients without prior cardiac surgery or documented atrial scarring is rare, as these complex circuits typically require anatomical obstacles rather than purely functional barriers. This case underscores that a "normal" right atrium can still harbor highly complex substrates capable of sustaining multiple reentrant loops. Dual-loop macroreentry may occur in non-surgical right atria due to functional conduction barriers. Prolongation of the tachycardia cycle length during ablation should raise suspicion of coexisting reentrant circuits rather than independent arrhythmias. Comprehensive high-density mapping and systematic entrainment are essential to identify all components of the tachycardia mechanism. CTI ablation alone may be insufficient when multiple right atrial loops coexist, and targeted substrate modification may be required to achieve durable termination.
